# New measurement technique for restoration of the trochlear offset after image-based robotic-assisted total knee arthroplasty: a reliability study

**DOI:** 10.1051/sicotj/2023027

**Published:** 2023-09-28

**Authors:** Moussa Kafelov, Jawhara Farhat, Elvire Servien, Sébastien Lustig, Cécile Batailler

**Affiliations:** 1 Orthopaedics surgery and Sports Medicine Department, FIFA medical center of excellence, Croix-Rousse Hospital, Lyon University Hospital 69004 Lyon France; 2 Univ Lyon, Claude Bernard Lyon 1 University, IFSTTAR, LBMC UMR_T9406 69622 Lyon France; 3 University Multiprofile Hospital for Active Treatment and Emergency Medicine “N. I. Pirogov” Sofia Bulgaria; 4 LIBM – EA 7424, Interuniversity Laboratory of Human Movement Science, Université Lyon 1 Lyon France

**Keywords:** Total knee arthroplasty, Native trochlea, Image-based robotic-assisted system, Anterior compartment, Personalized alignment

## Abstract

*Introduction*: The new concepts in total knee arthroplasty (TKA) tend to improve the alignment and ligament balancing after TKA. Nevertheless, the assessment of the anterior compartment is difficult. The purpose of this study was to describe a new measurement technique of trochlear offset restoration on CT-scan after primary robotic-assisted TKA and assess its reliability and repeatability. *Method*: This monocentric study assessed the trochlear offset restoration on a CT scan after 20 robotic-assisted TKA. To evaluate the trochlear offset restoration, we measured the depth difference between the native and the prosthetic trochlea. Four sequential positions were assessed on the trochlea: at full extension, at 30°, 70°, and 90° flexion. For each of these positions, we compared the highest point of the lateral native condyle and the lateral prosthetic condyle, the highest point of the medial native condyle and the medial prosthetic condyle, the deepest point of the native trochlear groove and the prosthetic trochlea. Two independent reviewers performed the measurements to assess their reliability. To determine intraobserver variability, the first observer performed the measurements twice. *Results*: The mean age was 67.3 years old ± 8.3. Mean values of the trochlear offset restoration for the medial condyle, trochlear groove and lateral condyle were respectively: 1.0 mm ± 1.6, 1.1 mm ± 1.5, −2.7 mm ± 2.3 in full extension; −3.5 mm ± 1.7, −1.5 mm ± 1.7, −3.9 mm ± 3.9 at 30° flexion; −5.1 mm ± 1.8, 2.1 mm ± 2.7, −3.8 mm ± 1.8 at 70° flexion; 2.0 mm ± 1.4 and 3.1 mm ± 1.5 for the medial and lateral condyles at 90° flexion. The radiographic measurements showed very good to excellent intra-observer and inter-observer agreements with mean kappa values of 0.92 and 0.74. *Conclusion*: We present a novel measurement technique on CT scan for evaluating the restoration of the trochlear offset after TKA, demonstrating excellent inter and intra-observer reliability.

## Introduction

Total knee arthroplasties (TKA) tend progressively to be adjusted to each patient, particularly with personalized alignment development. This TKA personalization occurred to improve functional outcomes [1, 2]. Indeed, a neutral alignment philosophy for every patient aimed to decrease wear and loosening rather than restoring normal knee kinematics and function. A systematic review has shown that between 10 and 34% of patients have an undesirable pain outcome after a primary TKA [3]. This pain can arise from multiple reasons, such as component malpositioning, malalignment, knee instability, or poor restoration of the anterior compartment with overstuffing [4].

The personalized alignment improves the knee kinematics after TKA. However, their impact on restoring the anterior compartment remains uncertain [5, 6]. Modern arthroplasty data demonstrated more than 15% of patients suffered from clinically significant patellofemoral dysfunction following TKA, even when the patella was resurfaced [7]. Alignment choice significantly affects the ability to restore the constitutional trochlea in TKA when using a standard femoral component [5]. Kinematic alignment allowed for restoring the constitutional trochlear groove, but a significant internal femoral rotation occurred in more than 25% of cases [8]. Functional alignment seems better for restoring the trochlea anatomy. Nevertheless, the main parameter analyzed in the literature for the anterior compartment is the positioning of the trochlear groove [8]. Indeed, the trochlear assessment in the literature was mainly with 2D radiographs with measurements of the femoral implant positioning in the coronal plane or with the posterior condylar offset in the sagittal plane. Assessment in 3D with CT scan was very uncommon with only the measurement of the trochlear groove orientation [5, 8]. The under or overstuffing of the anterior compartment remains little assessed. Nevertheless, under-stuffing has been shown to result in a reduction in the quadriceps lever arm [9] and overstuffing could lead to overhang, tightening the soft tissues, and limiting knee flexion and patellofemoral maltracking or anterior knee pain [10, 11]. The assessment of the trochlear offset restoration is thus primordial.

This study aimed to describe a new measurement technique assessing the restoration of the trochlear offset after image-based robotic-assisted TKA and to evaluate its reliability and repeatability. We hypothesized that this measurement technique was reliable with satisfying repeatability.

## Material and methods

### Study design

This retrospective monocentric study included 20 primary TKA performed by an image-based robotic-assisted system (MAKO robotic platform, Stryker, Mahwah, USA). Every patient had a varus deformity before the surgery (angle hip Knee Ankle (HKA) ≤ 183°). A specific CT scan with 3D reconstructions was performed before the surgery for every patient.

All surgeries were performed by two arthroplasty surgeons with more than five years of experience using robotic assistance for TKA and doing more than 200 cases yearly.

### Surgical technique

MAKO robotic platform planning software (Mako, Stryker Corp., Mahwah, NJ, USA) allowed preoperative implant planning using a patient-specific CT-based bone model and virtual implant templates. As previously shown, the 3D implant model followed the bony anatomy and had 1 mm of accuracy [12]. The CT scan-based preoperative planning using a three-dimensional (3D) model created by the software allowed individualized planning, implant sizing, and adjusting the position to suit the functional alignment principles. The positioning of the femoral implant respected the following principles: the coronal plane was included between 6° valgus and 3° varus, matching the lateral distal femoral angle (LDFA), the sagittal flexion plane was limited to 10° of flexion to avoid notching. The femoral rotation followed the posterior condylar axis (PCA) ± 3° and trans epicondylar axis (TEA) between 3° internal rotation and 6° external rotation. The femoral rotation was also adjusted on the shape of the native trochlea. The tibial component was placed respecting the medial proximal tibial angle (MPTA) with a limit of 6° of varus. The slope was limited to 3°, and rotation fitted Akagi’s line [13]. The implant sizing tried to respect the bone anatomy and avoid overhangs. The aim was to obtain balanced flexion and extension gaps of 17 mm.

### Measurement technique

All measurements were performed using MAKO robotic platform planning software (Mako, Stryker Corp., Mahwah, NJ, USA). A calibrated scale in millimeters allowed accurate and reliable measurements, with an accuracy of 1 mm. The measurements of the restoration of the trochlear offset after TKA were performed by two independent reviewers (an orthopedic surgeon and a medical student) for all measurements to assess the reliability of each measurement. To determine intraobserver variability, the second observer measured the patients twice, separated by a four-week interval. Both reviewers were trained on the MAKO platform to acquire the measurement technique.

**Figure 1:**
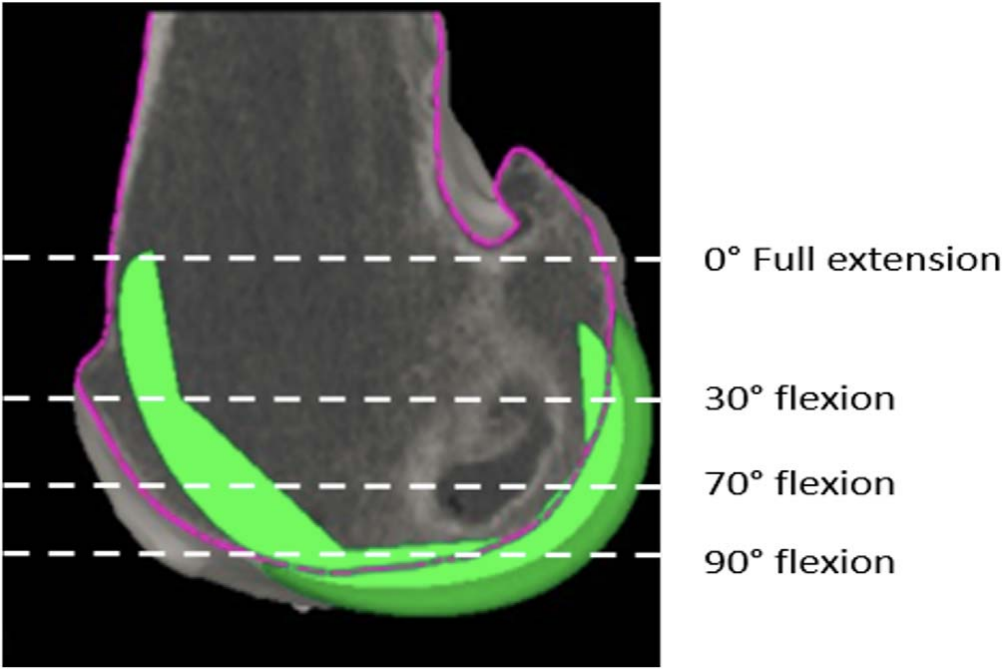
Measurement method – the difference between the native bone and the prosthesis was measured in four positions; full extension (0°), at 30° flexion, at 70° flexion, and in full flexion (90°). The position of the implant is in green. The purple outline indicates the patient’s native bony anatomy.

**Figure 2:**
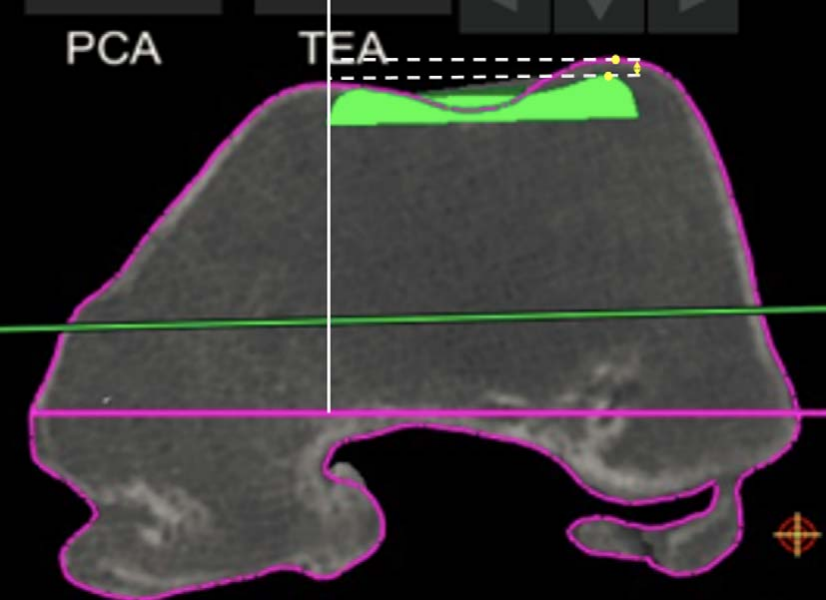
Measurement of the distance between the highest point on the prosthesis and the highest point on the patient’s native bone (lateral condyle full extension 0°).

**Figure 3:**
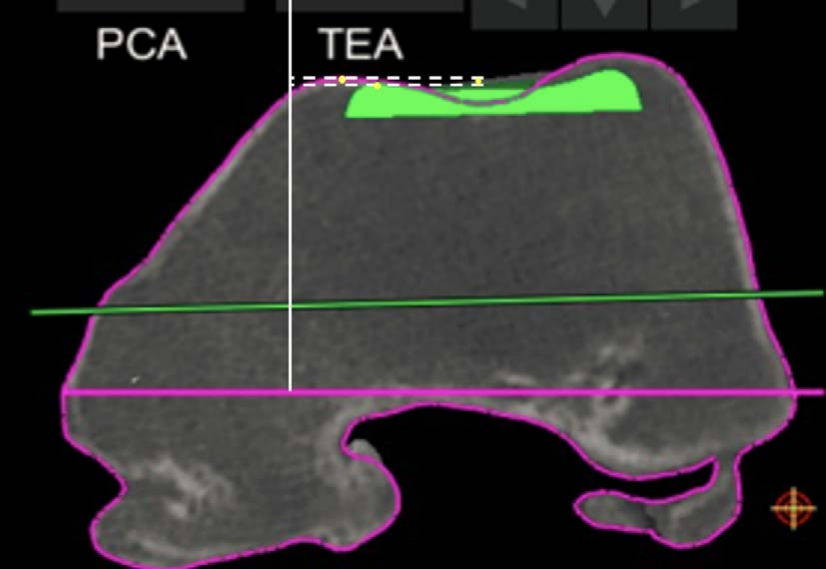
Measurement of the distance between the highest point on the prosthesis and the highest point on the patient’s native bone (medial condyle full extension 0°).

**Figure 4:**
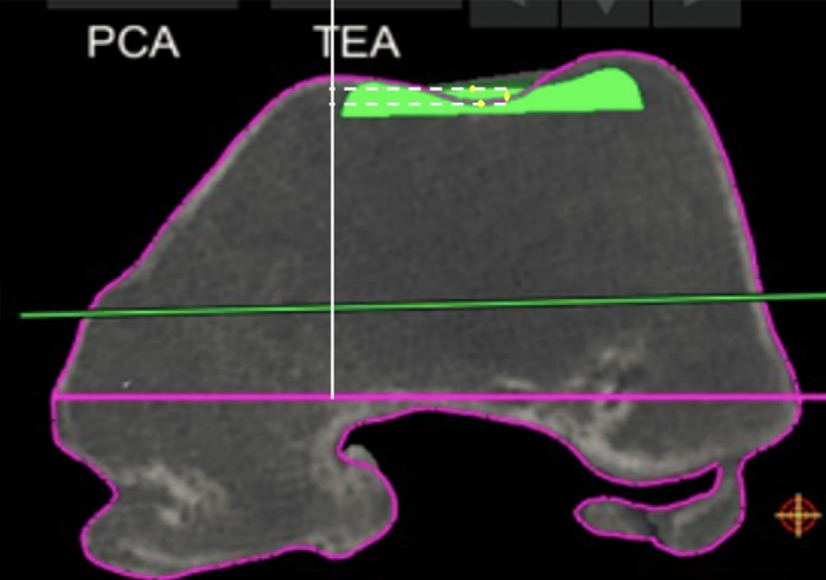
Measurement of the distance between the lowest point on the prosthesis and the lowest point on the patient’s native bone (Trochlea full extension).

For the trochlear offset, we measured the restoration of the trochlear thickness in several positions in the medial, lateral, and central parts of the trochlea. Four sequential positions were assessed at which the patella is engaged in the femoral groove with knee flexion: at full extension, at 30° flexion, at 70° flexion, and 90° flexion (Figure 1), as described previously [8]. For each of these positions, we choose an axial slice with the following markings: the first axial slice with a clearly defined prosthetic trochlear groove (“full extension”), the second was the merging point of the anterior chamfer and anterior flange on the sagittal view (“at 30° flexion”), the third one is the last slide on which both the prosthetic groove and femoral groove were visible (“at 70° flexion”), the fourth was the position on which the anterior flange and distal femoral part met (“at 90° flexion”). For each of these positions, we compared the highest point of the lateral native condyle and the lateral prosthetic condyle, the highest point of the medial native condyle and the medial prosthetic condyle, the deepest end of the native trochlear groove and the prosthetic trochlea (Figures 2–4). For these measurements, the reference line used was the trans-epicondylar axis.

### Statistical analysis

Statistical analysis was performed using the XL STAT software (Version 2021.2.1, Addinsoft Inc., Paris, France). The inter- and intra-observer reliabilities of the measurements were evaluated by an intraclass correlation coefficient. The strength of agreement for the kappa coefficient was interpreted as follows: < 0.20 = unacceptable, 0.20–0.39 = questionable, 0.40–0.59 = good, 0.60–0.79 = very good, and 0.80–1 = excellent [14].

## Results

The mean age was 67.3 years old ± 8.3 (50–84); the mean body mass index was 29.1 kg/m^2^ ± 4.1 (22.3–38.1); the mean HKA angle was 174.5° ± 3.8° (167°–182°).

The values of the trochlear offset restoration are summarized in Table 1.

**Table 1:** Values of the trochlear offset restoration according to the knee flexion and the localization.

Mean ± standard deviation (Minimum;Maximum)	Medial	Trochlear groove	Lateral
Full extension	1.0 ± 1.6 (−2;4)	1.1 ± 1.5 (−2;5)	−2.7 ± 2.3 (−7;3)
At 30° flexion	−3.5 ± 1.7 (−7;0)	−1.5 ± 1.7 (−5;2)	−3.9 ± 3.9 (−9;8)
At 70° flexion	−5.1 ± 1.8 (−8; − 2.5)	2.1 ± 2.7 (−4;6)	−3.8 ± 1.8 (−8; − 1)
At 90° flexion	2.0 ± 1.4 (0;4.5)	NA	3.1 ± 1.5 (1;5.5)

The radiographic measurements showed very good to excellent intra-observer and inter-observer agreements (Table 2).

**Table 2:** Intraobserver and inter-observer coefficients for the trochlear measurements.

	Intra observer ICC	Agreement	Inter observer ICC	Agreement
0° Medial	0.93	Excellent	0.82	Excellent
0° Lateral	0.79	Very good	0.64	Very good
0° Groove	0.64	Very good	0.60	Very good
30° Medial	0.96	Excellent	0.80	Excellent
30° Lateral	0.97	Excellent	0.86	Excellent
30° Groove	0.96	Excellent	0.82	Excellent
70° Medial	0.96	Excellent	0.76	Very good
70° Lateral	0.98	Excellent	0.83	Excellent
70° Groove	0.94	Excellent	0.69	Very good
90° Medial	0.98	Excellent	0.74	Very good
90° Lateral	0.96	Excellent	0.76	Very good

The strength of agreement for the kappa coefficient was interpreted as follows: < 0.20 = unacceptable, 0.20–0.39 = questionable, 0.40–0.59 = good, 0.60–0.79 = very good, and 0.80–1 = excellent.

## Discussion

The main finding of this present study was the description of a new measurement technique of the trochlear offset after total knee arthroplasty.

The choice of alignment philosophy led to significant variations in trochlear groove restoration. Functional and kinematic alignments equally recreate the trochlear groove in translation. However, kinematic alignment places the femoral coronal component in a position considered unsafe in 13.2% of cases, compared to functional alignment in 3.7% [8]. Neither KA nor MA techniques for TKA performed with conventional femoral implants restored native trochlea anatomy, stuffing, and orientation regardless of the approach and femoral component orientation [15]. A femoral component positioned by functional alignment principles most closely restored trochlear depth in all three positions of flexion.

Nevertheless, the trochlear offset and the stuffing of the anterior compartment are rarely precisely assessed. Data exploring the effect of alignment philosophy on over and under-stuffing of the trochlea is limited. An MRI-based study of 10 osteoarthritic knees demonstrated that kinematic alignment led to a mean understuffing of 5 mm in extension, 4.5 mm understuffing in mid-flexion, and component flush in 100° flexion [15]. Functional alignment tended to slightly overstuff the trochlear in full extension while under-stuffing through mid-flexion flexion [8]. In deep flexion, functional alignment resulted in a component flush with the native groove. By contrast, mechanical and kinematic alignments resulted in statistically more under-stuffing (1.0 mm and 2.2 mm, respectively). Trochlea depth restoration is complex with the use of a standard design implant. In biomechanical studies, under-stuffing has been shown to result in a reduction in the quadriceps lever arm [9]. Differences larger than 2 mm have been used to differentiate the native trochlea from overstuffing or under-stuffing [16, 17]. However, most of these measurements were performed on radiographs without high accuracy. Indeed, the radiographs cannot distinguish the medial and lateral parts of the trochlea or the trochlear groove. The overstuffing is frequently connected with shifting the trochlea groove anteriorly [17]. It could lead to overhang, tightening the soft tissues, and limiting knee flexion and patellofemoral maltracking or anterior knee pain [10, 11]. These patients are assumed to be more prone to limitations during their functional activities [18]. Though most of the studies on this subject are based on X-rays and mechanical instrumentation, the results are controversial, and it has certain limitations because of the methodology [19, 20].

This technique measurement allowed us to assess the restoration of the trochlear thickness with good accuracy and reliability. Several limitations should be outlined in this study and for this measurement technique. The main limitation of this study was the small number of patients. Nevertheless, it was enough to assess measurement techniques’ inter and intra-observer reliability. More patients should be considered for a clinical study to evaluate the correlation between the restoration of the anterior compartment and the functional outcomes. Second, we have assessed only one image-based robotic system with one implant. The extrapolation to other implants was not validated by this study.

The first limitation of this measurement technique was the necessity to use the MAKO system to measure the CT scan with the implants in place. Second, the measurement was based on a CT scan. Thus, it did not consider the cartilage thickness. To obtain the global restoration of the anterior compartment, it is necessary to add the thickness of the patella with and without the patellar button. Then, the four positions on the trochlea could be difficult to determine. However, with the same shape of the femoral implant, the landmarks were reliable, as demonstrated by the good inter-observer correlation.

To our knowledge, it is the first study describing a measurement technique assessing the restoration of the anterior compartment after TKA. This study did not aim to interpret the restoration of the trochlear offset, only to propose a reliable measurement technique.

## Conclusions

This new measurement technique assessed the restoration of the trochlear offset after TKA with good inter and intra-observer reliability. Another study should correlate the restoration of the anterior compartment after TKA with the functional outcomes.
